# The pleiotropic effects of α‐thalassemia on HbSS and HbSC sickle cell disease: Reduced erythrocyte cation co‐transport activity, serum erythropoietin, and transfusion burden, do not translate into increased survival

**DOI:** 10.1002/ajh.26652

**Published:** 2022-07-18

**Authors:** John N. Brewin, Amina Nardo‐Marino, Sara Stuart‐Smith, Sara El Hoss, Anke Hanneman, John Strouboulis, Stephan Menzel, John S. Gibson, David C. Rees

**Affiliations:** ^1^ Department of Haematological Medicine King's College Hospital London UK; ^2^ Comprehensive Cancer Centre, School of Cancer and Pharmaceutical Sciences King's College London London UK; ^3^ Centre for Haemoglobinopathies, Department of Haematology Copenhagen University Hospital (Rigshospitalet) Copenhagen Denmark; ^4^ Department of Veterinary Medicine University of Cambridge Cambridge UK

## Abstract

α‐Thalassemia is one of the most important genetic modulators of sickle cell disease (SCD). Both beneficial and detrimental effects have been described previously. We use a 12‐year data set on a large cohort of patients with HbSS (*n* = 411) and HbSC (*n* = 146) to examine a wide range of these clinical and laboratory associations. Our novel findings are that α‐thalassemia strongly reduces erythrocyte potassium chloride co‐transporter (KCC) activity in both HbSS and HbSC (*p* = .035 and *p* = .00045 respectively), suggesting a novel mechanism through which α‐thalassemia induces a milder phenotype by reducing red cell cation loss. This may be particularly important in HbSC where reduction in mean cell hemoglobin concentration is not seen and where KCC activity has previously been found to correlate with disease severity. Additionally, we show that α‐thalassemia not only increases hemoglobin in patients with HbSS (*p* = .0009) but also reduces erythropoietin values (*p* = .0005), demonstrating a measurable response to improved tissue oxygenation. We confirm the reno‐protective effect of α‐thalassemia in patients with HbSS, with reduced proteinuria (*p* = .003) and demonstrate a novel association with increased serum sodium (*p* = .0004) and reduced serum potassium values (*p* = 5.74 × 10^−10^). We found patients with α‐thalassemia had a reduced annualized transfusion burden in both HbSS and HbSC, but α‐thalassemia had no impact on annualized admission rates in either group. Finally, in a larger cohort, we report a median survival of 62 years in patients with HbSS (*n* = 899) and 80 years in those with HbSC (*n* = 240). α‐thalassemia did not influence survival in HbSS, but a nonsignificant trend was seen in those with HbSC.

## Graphical abstract

Alpha thalassemia has wide ranging effects in sickle cell disease. Most notably, it influences KCC co‐transport activity in both HbSS and HbSC disease. However, although there was a trend to improved survival in patients with HbSC, there was no effect on overall survival in patients with HbSS.

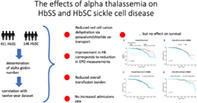

## 1 INTRODUCTION

Sickle cell disease (SCD) is a group of genetic disorders arising from a single point mutation in the hemoglobin subunit beta gene (*HBB*), leading to the formation of sickle hemoglobin (hemoglobin S, HbS). HbS can occur in homozygosity (HbSS), known as sickle cell anemia (SCA), or compound heterozygosity with complicit hemoglobin variants, most commonly HbC (forming HbSC). Despite this relatively uniform genetic origin, the clinical phenotype varies widely. Co‐inherited genetic variations are thought to contribute a significant element to this phenotypic diversity.

α‐Thalassemia is regarded as one of the primary genetic modifiers of severity in SCD.^1^ In people of Sub‐Saharan African descent, α‐thalassemia is most commonly caused by the 3.7‐kb α‐deletion denoted −α^3.7^. Other deletions, including −α^4.2^, and various small deletions and point mutations can also be seen; however, double α‐globin deletions α^−/−^ do not commonly occur. It is estimated that 30–35% of patients with SCD are heterozygous (αα/α−) and 3–5% homozygous for the deletion (α−/α−).^2^, ^3^, ^4^, ^5^

The beneficial effects of α‐thalassemia are thought to derive from the reduced concentration of hemoglobin in each erythrocyte, as measured by a lower mean cell hemoglobin concentration (MCHC), decreasing the tendency of hemoglobin S (HbS) to polymerize, leading to improved red cell deformability,^6^, ^7^, ^8^ decreased rates of hemolysis, and increased total hemoglobin values.^1^, ^7^, ^9^ The mechanism of detrimental effects is slightly more complex. The effect of the increased hematocrit on blood viscosity is balanced by the improved red cell deformability.^6^, ^10^ However, these more deformable red cells have been shown to be more adhesive and a rise in RBC aggregation is seen.^10^, ^11^ This likely promotes slower blood flow in the post‐capillary venules, leading to higher chances of a vaso‐occlusive episode (VOE) evolving.^7^, ^12^ It is well established that co‐inheritance of α‐thalassemia confers protection against cerebrovascular disease,^13^, ^14^, ^15^, ^16^, ^17^ including vasculopathy and ischemic stroke, as well as silent cerebral infarcts.^18^ It also plays a clear role in reducing renal complications,^19^, ^20^, ^21^, ^22^ priapism,^23^ and sickle leg ulcers.^24^ A protective effect against acute chest syndrome was initially reported,^5^ although findings have been inconsistent in subsequent studies,^25^, ^26^ with some studies finding an increased risk.^27^ The association with acute pain is similarly unclear. Some studies have found α thalassemia, particularly in the homozygous form,^10^, ^25^, ^26^, ^28^, ^29^, ^30^ to increase the frequency of VOE, whilst others studies have failed to find this.^31^, ^32^ Adverse effects of co‐inherited α‐thalassemia have also been identified. These include increased rates of avascular necrosis^33^, ^34^ and prolonged risk of acute splenic sequestration in childhood.^35^ Finally, some studies have suggested that α‐thalassemia can blunt the response to hydroxyurea (hydroxycarbamide) in patients with SCD,^36^ although others have not.^37^, ^38^

Co‐inheritance of α‐thalassemia in patients with HbSC leads to a milder phenotype, with a significantly reduced risk of acute splenic sequestration, reduced frequency of pain episodes, lower risk of osteonecrosis, and an overall improved survival rate.^39^, ^40^ Assessment of laboratory parameters has shown a reduction in mean cell volume (MCV), mean cell hemoglobin (MCH), reticulocyte percentage, white cell count (WCC), and lactate dehydrogenase (LDH), but no change in baseline hemoglobin (Hb), hematocrit, nor hemoglobin F percent (HbF%).^41^, ^42^ Hemoglobin C (HbC) is thought to contribute to intracellular HbS polymerization by encouraging red cell dehydration, in a way that hemoglobin A (HbA) does not.^43^ Thus, the red cell cation transport systems and maintenance of red cell hydration are thought to be a particularly important modulators of disease severity in HbSC‐disease.^43^

Three overactive transport mechanisms are primarily responsible for red cell dehydration in sickle erythrocytes: (1) the potassium chloride co‐transporter (KCC), which mediates obligatory coupled K^+^ and Cl^−^ efflux; (2) an ill‐defined cation conductance, referred to as P_sickle_, which is activated by deoxygenation, HbS polymerization, and red cell shape change; (3) the Gardos channel, a Ca2^+^‐activated K^+^ conductance, stimulated in particular by Ca^2+^ entry via P_sickle_. Solute loss via these transport systems causes RBC dehydration and elevation of the concentration of intracellular HbS leading to a greatly increased propensity to polymerize with a shorter lag time.^44^ Embury et al.^45^ demonstrated that the magnitude of the mean cation change upon deoxygenation of sickle red cells was directly related to α‐globin gene number.

In this study, we examined a large cohort of individuals with HbSS and HbSC‐disease to review the effects of α‐thalassemia on a wide range of clinical laboratory measurements taken at steady state in individuals treated both with and without hydroxyurea (HU) therapy. Second, we assessed the effects of co‐inherited α‐thalassemia on erythrocyte cation channel measurements. Finally, we considered annualized hospital admission rates and blood transfusion burden and performed survival analyses to determine any effect of α‐thalassemia on overall life expectancy in individuals with HbSS and HbSC‐disease.

## 2 METHODS

Patient data came from the South East London sickle gene bank cohort (London, UK). Written informed consent was obtained through approved study protocols (LREC 01‐083, 07/H0606/165, 12/LO/1610, 18/LO/1566, and 11/LO/0065) and research conducted in accordance with the Helsinki Declaration (1975, as revised 2008). Patients were recruited from five hospitals around southeast London. However, clinical laboratory data were only collected from the electronic patient record system at King's College Hospital. Only patients with the HbSS or HbSC genotype were included in this study.

### 2.1 Clinical data collection

Laboratory results from October 1, 2008 to October 1, 2020 were collected. Assimilation of blood transfusion history, hydroxyurea prescription, and in‐patient/emergency department visits allowed accurate contextualization of results. Steady‐state values were defined as >5 years from birth, in the outpatient setting, with no blood transfusion within 90 days, and free from pregnancy. Steady‐state values were further categorized as either on or off hydroxyurea. Where multiple values were available, average values were calculated, with the age defined as the average of age at which the laboratory tests were collected.

The annualized hospitalization rate, a measure of the number of visits per year, and the average number of inpatient days per year were calculated from the admission data. Patients were excluded from these measures if the duration of their follow‐up was less than 2 years, defined as the period from the earliest to the last recorded patient contact at the hospital between October 1, 2008 and October 1, 2020.

Survival data were collected using the earliest contact date with a Kings Health Partners (KHP) hospital as far back as January 1, 2000 as point of entry, and last contact with a KHP hospital, up to October 1, 2020, or date of death, as right truncation, or event censor, respectively. Patients were omitted if the duration of this follow‐up period was less than 1 year.

Red blood cell transfusion data were collected from the electronic patient records at KCH between October 1, 2008 and October 1, 2020. Any episodes where more than 6 units of blood were administered on the same day were assumed to be an exchange blood transfusion. Adherence to a regular red cell transfusion program was defined as regular top‐up transfusion (each within 45 days of the last) or regular exchange transfusion (each within 90 days of the last) or a combination of the two, stretching for at least six consecutive months. To calculate annualized transfusion rates, a minimum of 12 months of patient follow‐up was required. This was defined as the period from the earliest to the last recorded patient contact at the hospital. Two metrics were calculated for the eligible cohort of patients: average number of transfusion episodes per year and average number of red blood cell (RBC) units used per year.

### 2.2 Deletional α‐thalassemia genotyping

Single‐tube Multiplex PCR screening was performed according to previously published methods,^46^ with a combination of primers designed to detect the following deletions: −α.^3.7^, −α.^4.2^, —^SEA^, —^FIL^, —^MED^, and −α.^20.5^.

### 2.3 Red cell cation measurements

RBC samples were washed in simple 3‐(N‐morpholino) propanesulfonic acid (MOPS)‐buffered saline, comprising (in mM): 140 NaCl, 5 KCl, 1.1 CaCl2, 10 MOPS, 5 glucose, pH 7.4 at 37°C. Oxygen tension was controlled using a Wösthoff gas mixing pump with RBCs incubated in Eschweiler tonometers. RBC permeability was assessed using radioactive tracers (86Rb^+^) to measure the activity of the main cation transport systems involved in RBC dehydration: KCC, Gardos channel, and P_sickle_. KCC was measured as Cl^−^‐dependent K^+^ transport using NO3^−^ to substitute for Cl^−^. The Gardos channel was measured as clotrimazole‐sensitive K^+^ transport. P_sickle_ is defined as the deoxygenation‐induced Cl^−^‐insensitive K^+^ transport in the continued presence of clotrimazole. This method separates Gardos channel activity from that of P_sickle_. Assays were carried out in the presence of ouabain and bumetanide to exclude any contribution of flux via the other two main RBC cation transporters, the Na^+^/K^+^ pump and the Na^+^‐K^+^‐2Cl^−^ cotransporter. The concentration for the respective inhibitors was as follows: 5 mM for clotrimazole, 10 mM for bumetanide, and 100 mM for ouabain. For full details of methods, please see Hanneman et al.^43^

### 2.4 Statistical analysis

Logistic regression analysis, with gender and age of test included as covariates, was used to test the association of heterozygous and homozygous deletional α‐thalassemia against all clinical laboratory measurements for patients with either HbSS or HbSC genotypes. Laboratory measurements were all taken in steady state as defined above, and in the absence of concurrent hydroxyurea use. All quantitative variables underwent testing using the Shapiro–Wilks test of normality to consider whether logarithmically transforming the data improved its approximation of a normally distributed data set. If this was true, the variable measurements were logarithmically transformed before association analysis was performed. To account for the multiple testing of these parameters, the alpha level of statistical significance was set at .005 for all clinical laboratory measurements. Analyses of the effect on admission data, blood transfusion, red cell cation transporters, and responses to hydroxyurea were considered distinct data sets, therefore the threshold of significance was kept at the unadjusted level of .05. Paired *t* tests were used to assess patient responses to hydroxyurea, comparing measurements in the pre‐HU state with those during HU therapy for each patient. Unpaired *T* tests were used to determine whether co‐inheritance of α‐thalassemia affected the extent of these responses.

## 3 RESULTS

A total of 908 patients had a successful determination of their α‐globin numbers and were eligible for this study. Of these, 411 patients with HbSS, including 145 with aa/a− and 33 with a−/a− α‐thalassemia, and 146 patients with HbSC, including 45 with aa/a− and 2 with a−/a− α‐thalassemia, had steady‐state blood results available for analysis and were included in all further analysis, except for overall survival analysis, where the entire cohort was included. The average age of the patient cohort with HbSS was 34.8 years (range 7.5–78 years) whilst in those with HbSC, it was 45.1 years (range 15.4–80.5 years). Fifty‐four percent of the HbSS cohort and 62% of the HbSC cohort were female.

### 3.1 Red cell changes

As has been described previously, we found that α‐thalassemia was associated with a significant increase in total Hb in patients with HbSS, but not in those with HbSC. In both HbSS and HbSC, there was an overall increase in RBC count, and a concomitant reduction in absolute reticulocyte count (ARC) in individuals with co‐inherited α‐thalassemia (although the fall in ARC was not statistically significant after correction for multiple testing in the HbSS analysis) (Table 1). The hematocrit also significantly increased with decreasing α‐globin number in patients with HbSS, but not in patients with HbSC.

**TABLE 1:** Association analysis of co‐inheritance of α‐thalassemia in patients with sickle cell disease

A. Patients with HbSS					
Clinical parameters	Number of patients	αα/αα	αα/α−	α−/α−	*p*
Hb (g/L)	411	82.01 ± 13.47	84.08 ± 11.66	88.95 ± 10.37	**0.000877**
MCV (fL)	411	90.83 ± 9.35	82.81 ± 7.22	73.34 ± 4.57	**7.25 × 10** ^**−39**^
MCH (pg)	411	30.15 ± 3.44	26.96 ± 2.69	22.74 ± 1.39	**2.25 × 10** ^**−44**^
MCHC (g/L)	411	331.79 ± 13.18	325.52 ± 13.6	310.38 ± 12.9	**3.20 × 10** ^**−16**^
HCT (L/L)	411	0.25 ± 0.04	0.26 ± 0.04	0.29 ± 0.03	**7.30 × 10** ^**−08**^
RBC (×10^12^/L)	411	2.76 ± 0.58	3.16 ± 0.6	3.92 ± 0.5	**5.12 × 10** ^**−27**^
ARC (×10^9^/L)	404	379.16 ± 120.62	368.57 ± 106.86	321.59 ± 103.43	0.0149
Retic (%)	404	14.13 ± 4.58	12.04 ± 3.93	8.44 ± 3.16	**1.03 × 10** ^**−13**^
Platelets (×10^9^/L)	411	419.03 ± 127.24	394.68 ± 115.99	282.97 ± 99	**1.06 × 10** ^**−08**^
WCC (×10^9^/L)	411	10.45 ± 2.78	9.96 ± 3.06	8.96 ± 2.63	**0.000517**
Neutrophils (×10^9^/L)	409	5.46 ± 2.06	5.23 ± 2.05	4.85 ± 1.93	0.0717
Lymphocytes (×10^9^/L)	409	3.61 ± 1.05	3.48 ± 1.3	3.22 ± 1	**0.000973**
Eosinophils (×10^9^/L)	408	0.42 ± 0.54	0.31 ± 0.22	0.29 ± 0.2	**0.0022**
Monocytes (×10^9^/L)	409	0.77 ± 0.29	0.74 ± 0.4	0.55 ± 0.24	**1.12 × 10** ^**−05**^
HbF% (%)	411	7.44 ± 5.63	7.07 ± 4.81	6.47 ± 4.44	0.906
Erythropoietin (IU/L)	230	106.16 ± 78.42	92.74 ± 52.79	61.1 ± 28.86	**0.000508**
Potassium (mmol/L)	410	4.66 ± 0.45	4.46 ± 0.38	4.23 ± 0.33	**5.74 × 10** ^**−10**^
Sodium (mmol/L)	414	138.54 ± 1.78	138.92 ± 1.54	139.43 ± 1.16	**0.000413**
eGFR (mL/min/1.73^2^)	341	154.49 ± 46.46	165.58 ± 51.2	155.47 ± 48.02	0.479
uACR	358	10.67 ± 18.98	5.94 ± 9.92	5.79 ± 9.85	**0.00311**
LDH (IU/L)	391	521.84 ± 173.47	472.51 ± 153.26	389.82 ± 134	**2.79 × 10** ^**−07**^
Bilirubin (μmol/L)	404	65.52 ± 41.46	49.42 ± 30.26	28.44 ± 13.96	**5.49 × 10** ^**−15**^
AST (IU/L)	412	50.63 ± 28.43	44.71 ± 14.54	41.24 ± 12.46	**0.000553**
Gamma GT (IU/L)	414	61.52 ± 73.15	42.33 ± 42.63	53.29 ± 51.37	0.349
Albumin (g/L)	414	42.91 ± 3.52	43.81 ± 2.56	43.39 ± 2.17	0.115
CRP (mg/L)	332	12.11 ± 15.74	12.68 ± 16.93	7.33 ± 4.62	0.229
Admission per year	475	1.19 ± 1.42	1.31 ± 1.61	1.2 ± 1.52	0.658
Inpatient days per year	475	5.82 ± 10.37	6.08 ± 10.63	6.74 ± 11.8	0.881
Transfusion episodes per year	443	7.27 ± 17.02	4.66 ± 13.15	2.09 ± 5.05	**0.00826**
RBC units per year	443	0.65 ± 1.6	0.74 ± 2.18	0.18 ± 0.33	**0.0335**
P_sickle_ (K^+^ influx mmol/L cells h)^−1^	76	1.67 ± 0.57	1.68 ± 0.5	1.76 ± 0.84	0.7
Gardos (K^+^ influx mmol/L cells h)^−1^	76	5.07 ± 3.09	4.88 ± 2.69	5.26 ± 2.66	0.917
KCC (K^+^ influx mmol/L cells h)^−1^	76	3.28 ± 1.22	2.91 ± 1.11	2.53 ± 1.24	**0.0347**

B. Patients with HbSC					
Clinical parameters	Number of patients	αα/αα	αα/α−	α−/α−^a^	*p*
Hb (g/L)	146	114.83 ± 13.47	116.64 ± 11.66	114.34 ± 10.37	.451
MCV (fL)	146	79.59 ± 9.35	74.37 ± 7.22	68.87 ± 4.57	**9.61 × 10** ^**−07**^
MCH (pg)	146	27.64 ± 3.44	25.66 ± 2.69	23.63 ± 1.39	**4.51 × 10** ^**−07**^
MCHC (g/L)	146	347.03 ± 13.18	345.12 ± 13.6	342.73 ± 12.9	.251
HCT (L/L)	146	0.33 ± 0.04	0.34 ± 0.04	0.34 ± 0.03	.204
RBC (**×**10^12^/L)	146	4.18 ± 0.58	4.56 ± 0.6	4.88 ± 0.5	**6.64 × 10** ^**−06**^
ARC (**×**10^9^/L)	145	211.31 ± 120.62	177.94 ± 106.86	133.33 ± 103.43	**.00142**
Retic (%)	145	5.13 ± 4.58	3.97 ± 3.93	2.8 ± 3.16	**2.05 × 10** ^**−05**^
Platelets (**×**10^9^/L)	146	260.37 ± 127.24	222.93 ± 115.99	161.5 ± 99	.0377
WCC (**×**10^9^/L)	146	7.56 ± 2.78	6.41 ± 3.06	6 ± 2.63	.00717
Neutrophils (**×**10^9^/L)	146	4.19 ± 2.06	3.49 ± 2.05	3.58 ± 1.93	.016
Lymphocytes (**×**10^9^/L)	146	2.65 ± 1.05	2.33 ± 1.3	2.1 ± 1	.0849
Eosinophils (**×**10^9^/L)	145	0.22 ± 0.54	0.18 ± 0.22	0.09 ± 0.2	.373
Monocytes (**×**10^9^/L)	146	0.45 ± 0.29	0.36 ± 0.4	0.22 ± 0.24	.00686
HbF% (%)	146	1.84 ± 5.63	1.91 ± 4.81	2.55 ± 4.44	.302
Erythropoietin (IU/L)	118	41.37 ± 78.42	36.98 ± 52.79	43.31 ± 28.86	.193
Potassium (mmol/L)	147	4.3 ± 0.45	4.36 ± 0.38	4.15 ± 0.33	.52
Sodium (mmol/L)	147	139.74 ± 1.78	139.61 ± 1.54	140.59 ± 1.16	.979
eGFR (mL/min/1.73^2^)	104	115.66 ± 46.46	112.68 ± 51.2	117.65 ± 48.02	.674
uACR	135	3.18 ± 18.98	4.54 ± 9.92	2.32 ± 9.85	.821
LDH (IU/L)	145	264.11 ± 173.47	231.76 ± 153.26	181.28 ± 134	**.00039**
Bilirubin (μmol/L)	147	29.2 ± 41.46	23.04 ± 30.26	17.09 ± 13.96	.0132
AST (IU/L)	147	28.74 ± 28.43	28.52 ± 14.54	16 ± 12.46	.429
Gamma GT (IU/L)	147	37.35 ± 73.15	42.08 ± 42.63	20.71 ± 51.37	.905
Albumin (g/L)	147	43.25 ± 3.52	44.19 ± 2.56	43.03 ± 2.17	.0436
CRP (mg/L)	115	8.01 ± 15.74	7.04 ± 16.93	4.05 ± 4.62	.592
Admission per year	139	0.79 ± 1.42	0.6 ± 1.61	0.43 ± 1.52	.171
Inpatient days per year	139	3.09 ± 10.37	2.91 ± 10.63	0.85 ± 11.8	.144
Transfusion episodes per year	104	0.64 ± 2.04	0.04 ± 0.11	0 ± 0	.0617
RBC units per year	104	3.98 ± 13.29	0.1 ± 0.27	0 ± 0	**.0386**
P_sickle_ (K^+^ influx mmol/L cells.h)^−1^	70	1.07 ± 0.55	1.1 ± 0.71	0.73 ± 0.54	.373
Gardos (K^+^ influx mmol/L cells h)^−1^	70	2.78 ± 2.64	1.95 ± 1.9	2.41 ± 1.38	.213
KCC (K^+^ influx mmol/L cells h)^−1^	70	4.12 ± 1.25	3.19 ± 1.05	2.52 ± 1.55	**.00045**

*Note*: Age at time of test and gender were used as covariates in the analysis. *p* values in bold represent statistically significant results (after correction for multiple testing where appropriate, see methods for details)

^a^ α−/α− group contained only two patients and should not be used as a reference range.

Reductions in MCV and MCH were seen in individuals with co‐inherited α‐thalassemia in both HbSS and HbSC. However, only in patients with HbSS did the co‐inheritance of α‐thalassemia correlate with a fall in the MCHC.

Erythropoietin (EPO) measurements were available for 230 patients with HbSS and 118 patients with HbSC. These patients were not on EPO therapy at the time of the measurement. Reflecting on the changes seen with Hb, we found EPO values were lower in individuals with HbSS and co‐inherited α‐thalassemia, but not in patients with HbSC and co‐inherited α‐thalassemia. We found no influence of α‐thalassemia on HbF values in either cohort (Table 1).

### 3.2 Inflammatory and white cell count changes

There was a significant fall in total WCC in the HbSS cohort associated with the coinheritance of α‐thalassemia. However, there was no trend in neutrophil counts or CRP level. Instead, the reduction in WCC was due to reductions in lymphocytes, eosinophils, and most notably, monocytes (Table 1A). This trend was also seen in the HbSC cohort, however, after correction for multiple testing, was not statistically significant (Table 1B). We also found a significant reduction in platelet counts in individuals with co‐inherited α‐thalassemia. This effect was far stronger in the HbSS cohort, who had a higher platelet count overall, reflecting a heightened inflammatory state compared to those with HbSC.

### 3.3 Renal and cation changes

We found no association between CKD‐EPI‐derived estimated GFR and the number of α‐globin genes. However, we did observe a significant reduction in urinary albumin: creatinine ratio (uACR) values in the HbSS cohort *(p* = .003), but not in the HbSC cohort *(p* = .82) with α‐thalassemia. We also found plasma sodium and potassium values in the HbSS cohort varied significantly with α‐globin number, but not the HbSC cohort. Sodium values increased with each α‐globin deletion, whilst potassium values decreased (*p* = .0004 and *p* = 5.74 × 10^−10^, respectively, Table 1).

### 3.4 Red cell cation transport systems

**FIGURE 1:**
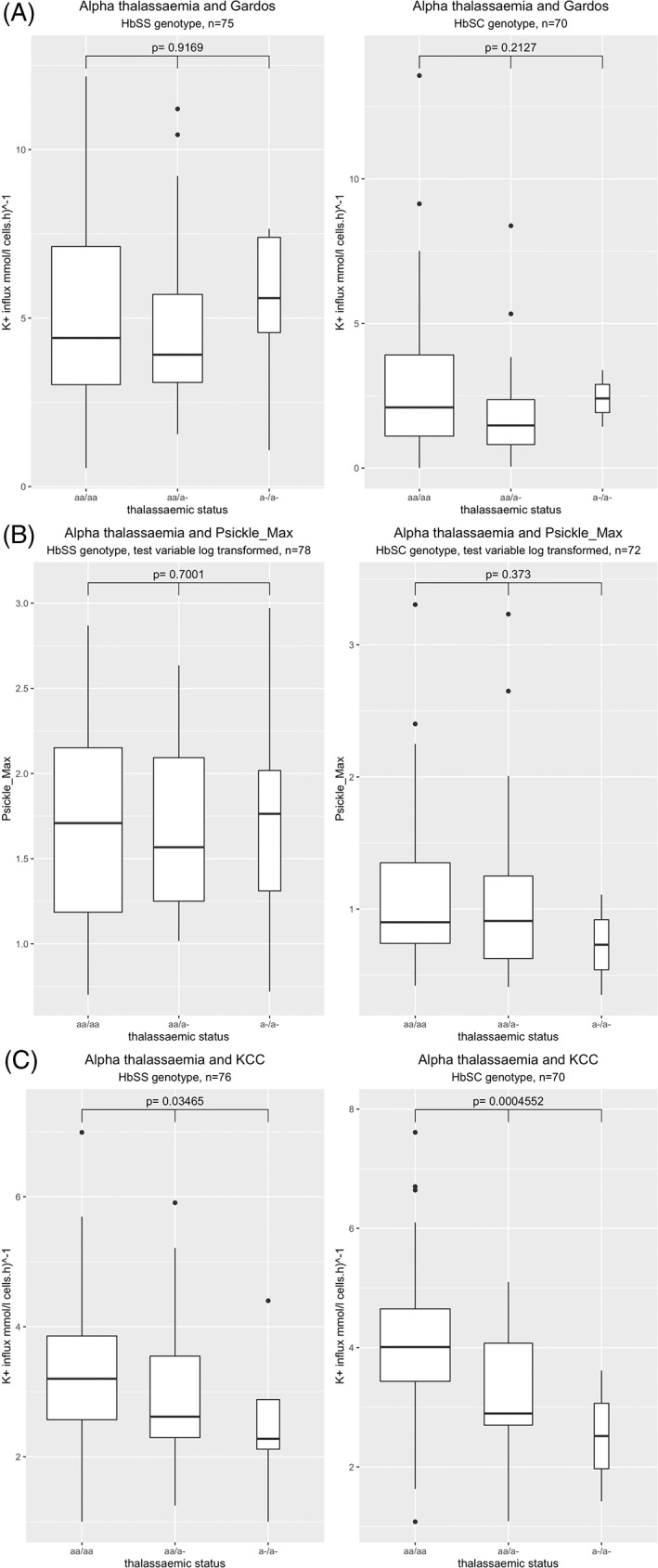
Box plots demonstrating the effect co‐inheritance of α‐thalassemia has on the three red cell cation transport systems: (A) Gardos channel; (B) P_sickle_; (C) potassium chloride co‐transporter (KCC)

Red cell cation measurements were available for 78 patients with HbSS and 72 with HbSC. P_sickle_ conductance or Gardos channel activity did not vary significantly with α‐thalassemia in either cohort (Figure 1). However, a significant decrease in KCC co‐transport activity in RBCs was seen in patients with α‐thalassemia in both the HbSS and HbSC cohorts, with a more marked effect seen in the HbSC cohort *(p* = .035 and *p* = .0005, respectively, Table 1).

### 3.5 Response to hydroxyurea therapy

This analysis focused on patients in the HbSS cohort, as there were insufficient numbers of patients with HbSC on hydroxyurea. One hundred forty individuals with HbSS had steady‐state blood results available both on and off hydroxyurea therapy, including 50 patients with aa/a−, and 11 with a−/a−. Paired *t* tests confirmed a statistically significant rise in HbF, Hb, MCV, and MCH and a statistically significant fall in MCHC across the 140 patients, demonstrating the expected response to hydroxyurea therapy across the cohort (Table 2).

**TABLE 2:** Effect of hydroxyurea therapy in patients with HbSS, and the effect co‐inheritance of α‐thalassemia has on the key changes seen

	Changes seen in cohort blood results before and during hydroxyurea therapy (*n* = 140)			Effect of α‐thalassemia on blood parameter changes		
	Mean of differences	95% CI	*p*	Beta coefficient	95% CI	*p*
HbF%	4.19	3.48–4.90	<2.20E−16	−0.63	−1.7 to 0.48	.268
Hb (g/L)	6.2	5.05–7.36	<2.20E−16	−1.4	−3.2 to 0.41	.132
MCV (fL)	13.74	12.15–15.33	<2.20E−16	−2.6	−5 to −0.11	.0428
MCH (pg)	4.07	3.56–4.07	<2.20E−16	−0.85	−1.6 to −0.052	.0387
MCHC (g/L)	−4.37	−6.21– −2.53	6.80E−06	0.43	−2.5 to 3.3	.773

When grouped by the number of α‐globin deletions, there was no evidence of a differential response in HbF and Hb increases, nor the MCHC reduction (*p* = .27, *p* = .13, and *p* = .77, respectively). There was, however, a moderate reduction in the degree to which MCV and MCH increased (*p* = .043 and *p* = .038, respectively) (Table 2).

### 3.6 Hospital admissions, blood transfusions, and overall survival

Four hundred seventy‐five patients with HbSS and 139 patients with HbSC had more than 2 years of follow‐up at KCH, accounting for 3833 and 1091 patient‐years, respectively. The average admissions per year was 1.23 and average number of inpatient days per year was 5.95 in patients with HbSS, whilst for patients with HbSC, the average admissions per year was 0.72 and average number of inpatient days per year was 3. We found no effect of α‐thalassemia on either admissions per year or inpatient days per year for patients with either HbSS or HbSC (Table 1).

Forty hundred forty‐three patients with HbSS and 104 patients with HbSC had sufficient information relating to transfusion history, accounting for 3513 and 804 patient‐years, respectively. The average number of transfusion episodes per year in patients with HbSS was 1.67 and the average number of RBC units used per year was 6.3. We found both measures to be significantly reduced in patients with co‐inheritance of α‐thalassemia (*p* = .00826 and *p* = .035, respectively, Table 1A). However, when we removed those on a regular transfusion program, there was no difference seen (*p* = .184 and *p* = .39). Moreover, when we removed only those on a transfusion program due to cerebrovascular complications, there was again no statistically significant difference.

The average number of transfusion episodes per year in patients with HbSC was 0.45 and the average number of RBC units used per year was 2.8. We found both measures to be reduced in patients with co‐inheritance of α‐thalassemia, but only the reduction in RBC units per year was statistically significant (*p* = .062 and *p* = .039 respectively, Table 1B).

**FIGURE 2:**
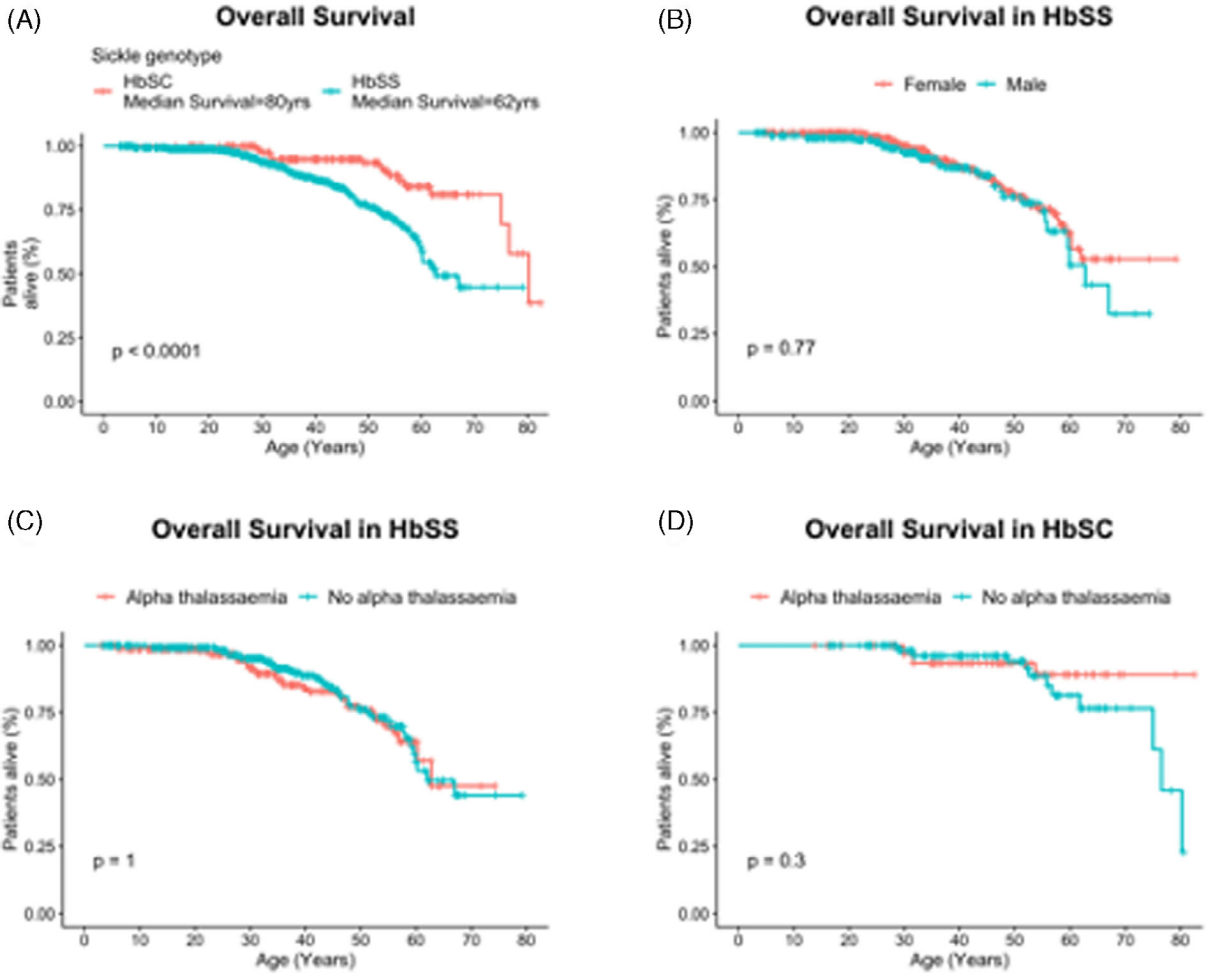
Survival analysis of patients with sickle cell disease. (A) Median survival analysis of all patients with HbSS and HbSC; (B) comparison of survival across gender in patients with HbSS; (C) the influence of α‐thalassemia on survival in patients with HbSS; (D) the influence of α‐thalassemia on survival in patients with HbSC [Color figure can be viewed at wileyonlinelibrary.com]

All 908 patients in the cohort were potentially able to contribute to the survival analysis, although nine were excluded because follow‐up was less than 1 year. The remaining 899 patients accounted for 14 662 patient‐years of observation and a mean follow‐up of 16 years. A total of 692 patients had HbSS from whom there were 68 deaths at an average age of 41.5 years. Two hundred seven patients had HbSC and there were 14 deaths in this cohort at an average age of 52.5 years. The median survival for these two patient groups was 62 and 80 years, respectively (Figure 2A), and we found no effect of gender on survival in either cohort (Figure 2B). When considering the influence of co‐inheritance of α‐thalassemia, no survival effect was seen in the HbSS cohort (Figure 2C). However, in the HbSC cohort, there was a trend toward improved survival with fewer α‐globin genes, although this did not reach statistical significance (Figure 2D).

## 4 DISCUSSION

This study considers the various effects of α‐thalassemia in a large cohort of individuals with HbSS and HbSC‐disease. We confirm many well‐described effects of α‐thalassemia, such as that on red cell indices, total Hb, and markers of hemolysis, and go on to identify a number of important novel observations.

We found that co‐inheritance of α‐thalassemia significantly decreased the activity of the KCC co‐transporter, an effect that was most notable in patients with HbSC. This co‐transporter is markedly upregulated in sickle red cells, particularly in patients with HbSC disease^43^ and is thought to be an important driver of red cell dehydration. The mechanism by which α‐thalassemia induces a milder phenotype in HbSC^39^, ^40^ has so far been poorly understood. By demonstrating a significant reduction in KCC activity in the red cells of patients with HbSC, we have identified a novel biological mechanism by which α‐thalassemia may be acting in these patients to improve the cation content of the red cell and reduce the rate of hemoglobin polymerization. This concurs with our previous observation that lower KCC activity is associated with fewer clinical complications in HbSC disease.^47^ This effect on KCC co‐transporter activity was also seen in the HbSS cohort. The reduction in red cell dehydration is likely to be an important mechanism of the anti‐sickling effect of α‐thalassemia and may contribute to the improved rheological characteristics of sickle erythrocytes where α‐thalassemia is present.^6^, ^8^ Exactly how co‐inheritance of α‐thalassemia and sickle Hb results in a reduced activity of KCC is an important question. KCC is regulated by protein phosphorylation involving a cascade of protein kinases and phosphatases.^48^ It also responds to cell volume, probably via effects on macromolecular crowding, and also to other modalities such as oxygen tension and pH.^48^ Some of these involve interactions between hemoglobin and the cytoplasmic tail of band 3 (the anion exchanger) and have been shown to affect phosphorylation status of the red cell cotransporters.^49^ We speculate that reduced levels of alpha chain impact one or more of these pathways, ultimately increasing phosphorylation of a key regulatory step leading to reduction in KCC activity. It is also possible that KCC activity is related to the rate of HbS polymerization in an undefined way, and that the change in KCC activity is caused by the reduced rate of polymerization associated with α‐thalassemia. Addressing these postulates will likely require careful analysis of the red cell phosphoproteome.

This study also confirmed the protective influence of α‐thalassemia on renal function in patients with HbSS.^19^, ^20^, ^21^, ^22^ Due to the initial hyperfiltration seen in children with SCD,^50^ from which the decline of eGFR takes a long time to become significant according to normal thresholds, eGFR is a poor marker of renal disease in this type of analysis. Instead, uACR is one of the clearest markers of early and ongoing renal dysfunction. We found a significant association between reduced values of uACR and α‐thalassemia in patients with HbSS, but not HbSC. To minimize bias, all measurements were taken from patients when hydroxyurea was not being prescribed, which is reported to improve proteinuria values.^51^, ^52^ Our data does not control for use of Acetyl Choline Esterase inhibitors (ACEi), which can reduce uACR values,^51^ or concomitant type 2 diabetes mellitus, which is known to affect renal function and uACR independently of SCD. However, the risk of developing T2DM is not known to be influenced by α‐thalassemia, and therefore should affect both groups equally, and, by taking averages of the measured values over 12 years (rather than simply the last measurement, which may have significantly reduced again following ACEi therapy), results should strongly indicate those who had elevated values versus those without.

In addition, we found that serum potassium and sodium values were significantly affected in the HbSS cohort. α‐Thalassemia was associated with higher sodium and lower potassium values. An isolated decrease in potassium values may have been an artifact due to less potassium leaking from red cells ex‐vivo prior to analysis in those with α‐thalassemia; however, the concomitant rise in sodium values suggests that this is may be a reflection of renal function. Drawz et al.^53^ found rising serum potassium to be associated with worsening eGFR in patients with HbSS, and our findings would be in keeping with that. The range of potassium and sodium values remained within normal limits, and it is likely that this is simply a marker of renal dysfunction, rather than a clinical parameter to aim to control therapeutically.

Our survival analysis included a cohort of almost 900 patients, one of the largest to date. We accounted for the left truncation bias that was a criticism^54^ of the previously published analysis from our institution. It is important to observe that this is indeed a more conservative estimate of median survival in patients with HbSS at 62 years (previously 67 years^55^), but still higher than the US estimate of 48 years, and markedly higher in our HbSC cohort at 80 years versus 54.7 years in the US analysis.^54^ The reasons for such a discrepancy are not clear but may reflect the differences in access to all aspects of health care in the different countries. We observed no gender discrepancy in the survival analysis. This finding was in keeping with other recent studies,^56^ although in contrast to the original co‐operative study of sickle cell disease analysis.^57^ We found no influence of α‐thalassemia on survival rates in patients with HbSS, but a trend to improved survival in those with HbSC. Probably the small number of deaths in this cohort limited the statistical power of the analysis. Other limitations of this analysis are that it uses all‐cause mortality, and not just those deaths directly related to SCD. Second, the cohort came from those who have consented to participate in trials over the last two decades and, as such, may be vulnerable to a selection bias. We also only had two patients with HbSC and homozygous α‐thalassaemia, which limits the conclusions that can be drawn from this sub‐group in all the analyses.

Although admission rates are recognized to be a poor surrogate marker of VOE, we found no influence of α‐thalassemia on average of inpatient days per year, or annualized admission rates. This is an important finding, as many studies report that patients with co‐inheritance of α‐thalassemia and HbSS experience increased frequency of VOE.^10^, ^25^, ^26^, ^28^, ^29^, ^30^ It is not entirely clear why such discrepancy continues to be reported. Of note, those that report higher VOE rates are more typically commenting on the homozygous α‐thalassemia population (α−/α−) and therefore have very small sample sizes. We only had 33 such patients, this is significantly more than most other studies. However, our analysis may also be undermined using acute hospital admission as a surrogate for VOE. We may be missing many outpatient‐managed VOE events, or equally conflating other reasons for acute admission with VOE events. Importantly, however, we show the evidence for increased VOE in patients with α‐thalassemia is not strong and needs to be interpreted with caution. We also showed that co‐inheritance of α‐thalassemia reduced transfusion requirements in both HbSS and HbSC patients, but that in those with HbSS, this appeared to be primarily through a reduction in the number of patients committed to long‐term transfusion programs, and further analysis suggested that this is mostly due to the known reduction in the cerebrovascular complications which necessitate a long‐term transfusion program.

As expected, we found that in patients with HbSS, but not HbSC, baseline Hb was elevated in a stepwise fashion with α‐thalassemia. Additionally, we found that this was accompanied by a fall in EPO values. This is likely a response to improved tissue oxygenation reducing erythropoietic drive, although this has not previously been shown, and shows that a relatively small increase in hemoglobin results in a significant fall in erythropoietin values. This evidence may have important implications for emerging therapies that, through influencing the hemoglobin oxygen affinities, result in elevated Hb. The concern regarding such irreversible modulation is that tissue oxygenation is not significantly improved because the modified Hb does not offer up its oxygen molecules in physiological conditions,^58^ leaving the patient functionally more anemic. If such therapies were able to demonstrate a concomitant fall in erythropoietin, this would provide a reassuring marker of improved tissue oxygenation.^59^

Finally, we report no clinically significant difference in the response to HU between patients with or without α‐thalassemia. We found an attenuated increase in MCV and MCH associated with starting HU, but no difference in the changes to HbF, total Hb, or MCHC. This result is in keeping with analysis from the MSH^37^ and BABY‐HUG^38^ studies, although other smaller studies have reported contrasting findings.^36^ In keeping with a recent publication,^60^ we also demonstrated that treatment with HU precipitated a fall in MCHC, and that this was still seen in those with α‐thalassemia despite the lower baseline MCHC. This likely represents an important therapeutic effect of HU in addition to its primary effect of raising the HbF%.

In summary, we have used a large patient cohort with well‐defined α‐thalassemia status to review the many effects that this co‐inherited genetic trait has on outcomes in patients with HbSS and HbSC‐disease. Our most salient finding is the effect seen on the red cell KCC co‐transporter system, with particular emphasis on the role this may have in the protective effect of α‐thalassemia in HbSC disease. Second, our survival data do not suggest a marked survival advantage associated with α‐thalassemia and confirm that the median survival is beyond 60 years in HbSS in the UK. We have also shown that α‐thalassemia is associated with less renal impairment and reduced values of proteinuria and serum potassium. Overall, α‐thalassemia has a significant effect on many parameters in SCD and should be considered in the interpretation of clinical complications and laboratory results of all patients. It should also be considered in the design of clinical trials in SCD, particularly in studies looking at changes in renal function or hemoglobin values.

## AUTHOR CONTRIBUTIONS

John N. Brewin and David C. Rees designed the study. John N. Brewin, Amina Nardo‐Marino, Sara Stuart‐Smith, Sara El Hoss, John Strouboulis, Anke Hanneman, John S. Gibson, and Stephan Menzel recruited patients and performed research. John N. Brewin analyzed the data and wrote the manuscript. All authors reviewed and approved the manuscript prior to submission.

## CONFLICT OF INTEREST

The authors declare no conflict of interest.

## Data Availability

The data that support the findings of this study are available from the corresponding author upon reasonable request.
